# Natural Versus Vaccine-Induced Immunity Against HPV: A Comparative Review of Antibody Response and Cancer Prevention

**DOI:** 10.3390/antib15040061

**Published:** 2026-07-13

**Authors:** Bogdan Ghilencea, Bianca Ilinca Moroianu, Ancuța-Iuliana Năstac, Ioana-Stefania Bostan, Anca Panaitescu, Claudia Mehedințu, Nicolae Gică

**Affiliations:** 1 Faculty of Medicine, “Carol Davila” University of Medicine and Pharmacy, 050471 Bucharest, Romania; bogdan.ghilencea2022@stud.umfcd.ro (B.G.); ioana-stefania.bostan@rez.umfcd.ro (I.-S.B.);; 2 Department of Obstetrics and Gynecology, Filantropia Clinical Hospital, “Carol Davila” University of Medicine and Pharmacy, 050474 Bucharest, Romania

**Keywords:** HPV, immunity, vaccination, antibodies, cancer prevention

## Abstract

Human Papillomavirus (HPV) infection is the leading cause of cervical cancer, presenting a significant global health challenge. While natural infection is widespread, the resulting immune response is often characterized by weak, delayed, and type-specific antibody production, offering unreliable protection against reinfection. This review provides a comparative analysis of natural versus vaccine-induced immunity, focusing on antibody kinetics, duration of protection, and cancer prevention efficacy. A comprehensive search of the literature from the last decade was conducted using PubMed, ScienceDirect, and Web of Science. The findings demonstrate that, unlike natural immunity, which is dominated by cellular responses with often incomplete seroconversion, prophylactic vaccination induces high titers of neutralizing IgG antibodies against the L1 capsid protein. These responses are durable, with protection persisting for over a decade, and recent data support the high efficacy of single-dose regimens. Furthermore, vaccination has shown utility in reducing infection persistence in HPV-positive individuals and provides critical protection in immunocompromised groups. Consequently, vaccine-induced immunity is consistently superior to naturally acquired immunity, supporting World Health Organization recommendations for universal vaccination as the primary intervention for reducing the global burden of HPV-related malignancies.

## 1. Introduction

Human papillomavirus (HPV) is the most common sexually transmitted infection worldwide, affecting nearly 80% of sexually active individuals at least once in their lifetime [1,2]. High-risk genotypes, especially HPV-16 and 18, are responsible for a majority of HPV-related cancers, including cervical, anal, penile, and oropharyngeal malignancies. Epidemiological studies show marked sex differences, with men experiencing higher rates of anogenital HPV infection and rising HPV-driven oropharyngeal cancer incidence, with a lower global cancer risk [2]. Furthermore, HPV DNA has been detected both in women with abnormal cervical cytology and healthy women, with the frequency of HPV-positive specimens rising with the severity of cervical lesions [3,4]. Despite the availability of highly effective vaccines, global coverage remains suboptimal, especially in low- and middle-income countries (vaccination coverage is at about 12–15%) [2].

With over 200 distinct genotypes, which are classified as high-risk or low-risk based on their oncogenic potential, HPV infections are extremely diverse [5]. High-risk HPV types are predominantly associated with cervical and other anogenital cancers [6]. HPV types 16 and 18 are the most oncogenic, collectively responsible for over 70% of cervical cancer cases worldwide [5,7]. Other high-risk types, including HPV 31, 33, 45, 52, and 58, also contribute to cervical carcinogenesis, though less frequently [8,9]. HPV 16, in particular, has been extensively studied and is known to cause the majority of HPV-related cervical malignancies [5]. In contrast, low-risk HPV types, such as HPV 6 and 11, are mostly linked to benign lesions such as genital warts and are rarely associated with malignancy [8].

The critical factor that increases the risk of cervical cancer is the persistent infection with oncogenic HPV types, leading to genetic instability in cervical epithelial cells [10]. The main mechanism is via viral genome integration in the host cells’ DNA, ultimately leading to malignant transformation [11,12].

Given the widespread prevalence of human papillomavirus (HPV) infection, a key question concerns the extent to which natural immunity provides protection comparable to that induced by vaccination [13]. The immune response to HPV infection differs markedly between naturally acquired and vaccine-induced immunity, with significant implications for the duration of protection, antibody titers, and cancer prevention efficacy [14].

Natural immunity to HPV infection generally exhibits a weak and type-specific antibody response [15]. The immune system’s feedback to natural HPV infection often involves a delayed, low-titer production of neutralizing antibodies, primarily directed against the viral L1 protein [14].

## 2. Materials and Methods

**Figure 1:**
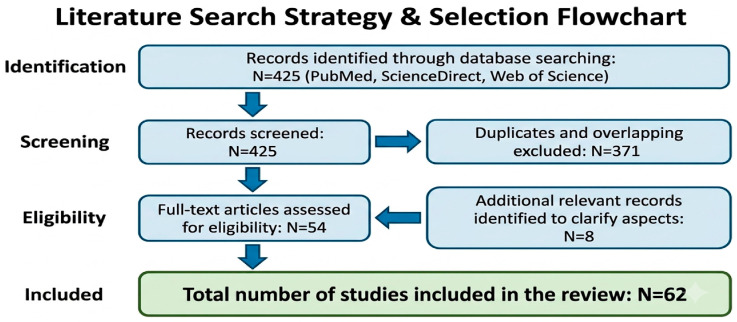
PRISMA-style flowchart detailing the literature selection process for the narrative review. The diagram outlines the phases of identification (*n* = 425), screening and duplicate exclusion (*n* = 371), eligibility assessment of full-text articles (*n* = 54), inclusion of supplementary records to address specific gaps (*n* = 8), and the final pool of synthesized studies (*n* = 62).

This narrative review was conducted through a comprehensive literature search using the PubMed, ScienceDirect, and Web of Science databases. The search strategy included the following keywords and Boolean combinations: ‘HPV’, ‘natural antibodies’, ‘vaccine-induced antibodies’, ‘cervical cancer’, ‘antibody titer’, and ‘protection. The inclusion criteria consisted of original research articles available in full text, and relevant to the topic of HPV immunity or protection. Only studies from the last 10 years were selected, as we considered it more relevant to bring forth the most recent data and present the novelties in the field. Initially we reviewed 425 articles, of which 371 were either overlapping or duplicates, therefore they were excluded. To the remaining 54 selected articles, an additional 8 relevant studies were added to clarify incomplete aspects, resulting in a total of 62 included articles. This process was summarised in Figure 1.

While a total of 64 articles were included in the qualitative synthesis to provide a comprehensive overview of the field, a summary of the most prominent studies explicitly detailed in this review, highlighting their methodologies, cohort sizes, and geographical locations is presented in Table 1.

The aim of this analysis was to compare the immune response and level of protection provided by natural immunity after HPV infection with those induced by vaccination, focusing on antibody titers, duration of protection, and efficacy in preventing HPV-associated cancers.

## 3. Results

The data are presented in two main parts: natural immunity following HPV infection and immunity acquired through vaccination. This division allows comparison of how the body responds to infection versus to prophylactic vaccines. However, interpretation of antibody titers remains difficult, as protocols for measurement and protective cut-off values vary across studies, with IU/mL being the most widely used unit.

### 3.1. Natural Immunity After HPV Infection

Natural immunity acquired after HPV infection is dominated by cellular responses, with slow and often incomplete seroconversion [33,34]. Only a small percentage of infected subjects develop measurable titers of neutralizing antibodies, and these remain low, offering limited protection compared with vaccine-induced immunity. The following section summarises data regarding cellular responses, antibody profiles, and seroprevalence data from representative cohorts.

At the cervical epithelium level, some studies identified several types of inflammatory cells, including CD16+ granulocytes, γδ T cells, CD4+ and CD8+ lymphocytes [33,34]. Other cell types include APCs and NK cells. These cells are important for local infection control through cytokine secretion (e.g., CXCL10), which promotes chemotaxis and T-helper lymphocytes development. HPV can blunt this response, notably by MHC downregulation mediated by E6/E7 proteins, at the same time reducing the humoral response.

Beyond cellular immunity, antibodies play an important role in countering infection, involving IgA, IgM, and IgG classes [35]. Current consensus underlines IgG neutralising antibodies, directed against the L1 capsid protein, as the most reliable protective marker. The total level of antibodies is less informative in regard to protection [35].

#### Seroprevalence and Protection: Evidence from International Studies

To assess the effectiveness of natural immunity, it is essential to evaluate the seroprevalence of anti-HPV antibodies in unvaccinated populations and their correlation with protection against persistent infections. The following studies provide evidence gathered in large international cohorts.

A. A large stage three study [7] followed seroconversion for HPV 16/18 in two cohorts of unvaccinated women over the period of four years. Of the 10,752 women screened for HPV-16 antibodies, only 18% (1935 women) were positive for the neutralising IgG. In the HPV-18 cohort (11,169 women), 15% (1675 cases) showed neutralizing IgG seropositivity [7]. However, clinical outcomes underlined the limited protective value of natural immunity: 1607 lesions progressed toward malignancy (mostly HPV-16 related), with 37% incidence of newly acquired infections, 30% persistent infections lasting over two years, and 43% squamous cell atypia [7]. These results indicate that naturally acquired antibodies are insufficient to prevent disease progression, particularly for HPV-16.
B. A Dutch cohort study [36], which involved several thousand women near menopause, assessed the relation between naturally acquired HPV antibodies and protection against subsequent infections. Although women with detectable antibodies were expected to be less vulnerable to new HPV infections, the data did not show a statistically significant difference. However, those same antibodies did appear to offer better protection against persistent infections, which are more likely to lead to malignant changes [36]. In this cohort, 30% of participants had detectable anti-HPV 16/18 antibodies, yet only 9.95% of them reported an HPV infection history [36]. These results indicate that although natural immunity may help limit long-term viral persistence, it does not reliably prevent initial infection.
C. In a cohort of 978 unvaccinated women from China [37], researchers measured levels of anti-HPV 16/18 antibodies—both neutralizing IgG and total IgG (including binding, non-neutralizing antibodies). With high statistical significance (*p* < 0.05) and strong data correlation (k between 0.52 and 0.38), their results underline that neutralizing IgG antibodies are the most important markers of protection against persistent HPV infections [37].
D. A 2023 Canadian study assessed the seroprevalence of HPV antibodies in 222 unvaccinated women [38], with results in close agreement with other findings as evidenced before. The distribution of seropositivity across HPV strains is shown in Table 2 [38].

The researchers used glutathione-S-transferase purification followed by affinity-based methods to detect and analyse antibody profiles [38]. Their findings showed clear patterns of antibody type quantity and relevance to malignancy prevention, summarized in Table 3.

These results reinforce that while IgG anti-L1 is most abundant, antibodies against E6/E7 are more relevant for malignancy prevention, in line with previous findings.

E. Important data are provided by two studies conducted in China. The first study [39] followed women with and without HPV infection through periodic cytological examinations and serum antibody measurements. Among HPV-positive women, infection was most often caused by a single strain, with coinfections being rare (although one case of simultaneous infection with HPV types 16, 18, 33, and 45 was documented) [39]. Antibody titres were significantly higher in HPV-positive women, particularly in those with lesions (e.g., four women with CIN III). Interestingly, 45% of women without demonstrable infection also had detectable anti-HPV antibodies, predominantly against types 16 and 18 [39].
F. The second study [40] used a similar approach, measuring antibodies and performing cytological evaluation in 1897 women with CIN. The most notable finding was the higher prevalence of antibodies against non-high-risk HPV types, which may explain the relatively limited protection observed after natural infection. It is important to note that these studies measured total antibodies, not specifically neutralizing antibodies [40].
G. Another important aspect of HPV immunity is the possibility of antibody transfer through the placental barrier. A 2022 Finnish study [41] analyzed serum samples from 276 mother–infant pairs, from right after birth to the 36-month mark. They measured antibodies for the 6, 11, 16, 18, and 45 HPV strains, and identified two distinct pathways in which they evolve. The first reflects passive maternal transmission: at birth, 40–62% of newborns had detectable anti-HPV antibodies, a finding strongly correlated with maternal seropositivity (*p* < 0.001) [41]. These passively acquired antibodies declined steadily, becoming negligible by 8–10 months, consistent with the natural fading of maternal immunity as antibodies were metabolized. The second pathway points to independent early contact. By 36 months, 53% of children born to seronegative mothers had developed anti-HPV antibodies, most likely through early environmental contact with the virus via fomites or caregivers [41]. The results indicate that maternal immune antibodies provide only short-term passive protection, while early postnatal exposure can independently induce antibody responses in children.
H. Natural immunity against HPV is weak and often inconsistent [7,42]. The cellular immune response plays a central role, yet seroconversion is inefficient, and neutralizing antibody titers remain low. Studies regularly emphasize the importance of IgG neutralising antibodies (especially anti-L1) as a protective marker. Yet the absence of an international standard for cut-off values complicates standardisation. Passive transmission across the placental barrier offers temporary protection; however, initial exposure to HPV maintains its status as a genuine public health concern. This evidence emphasizes the limited and unreliable nature of natural immunity, supporting the need for vaccination as the primary strategy for durable protection.

### 3.2. Acquired Immunity After Vaccination

In the case of vaccine-induced immunity, the process is different. HPV vaccines have proven highly effective in avoiding both reinfections and persistent infections. The most striking difference between post-infection and post-vaccination immunity lies in antibody titers: vaccinated subjects develop significantly higher levels of neutralizing antibodies, translating into superior and more durable protection against HPV.

#### 3.2.1. Types of HPV Vaccines

The vaccines currently available against human papillomavirus infection are produced using recombinant DNA technology. This process involves inserting the gene encoding the HPV L1 capsid protein into host cells to produce the protein in large quantities. These proteins naturally assemble into virus-like particles (VLPs) that closely resemble the structure of the actual virus but do not contain viral DNA, making them non-infectious and safe. Once purified and combined with an adjuvant to enhance the immune response, these VLPs form the basis of the vaccine, allowing the body to recognize and fight HPV without exposure to the actual virus.

Currently, three HPV vaccine formulations are available on the market: bivalent (Cervarix), quadrivalent (Gardasil 4), and nonavalent (Gardasil 9) [35,43]. Each vaccine offers protection by initiating an immune response through L1 virus-like particles (VLPs), which are structural mimics of the L1 capsid protein corresponding to each targeted HPV strain. Each formulation has a specific range of strains against which they offer protection. The bivalent vaccine protects against HPV-16 and HPV-18, while the quadrivalent formulation protects against HPV-6, HPV-11, HPV-16, and HPV-18 [43]. The nonavalent vaccine has the broadest protection spectrum, against the following strains: HPV-6, -11, -16, -18, -31, -33, -45, -52, -58 [43].

As mentioned above, there is an increase in the range of vaccine-induced protection from the bivalent formulations to the nonavalent ones. The latter covers more strains, especially high-risk ones, than any other counterpart [43]. Following interaction between L1 VLPs and immune cells, neutralizing IgG antibodies are generated, binding specifically to epitopes on the L1 capsid protein.

#### 3.2.2. Cross-Reactivity

Antibodies induced by vaccination against human papillomavirus infection may exhibit cross-reactivity, providing some protection against HPV types not specifically included in the vaccine. However, this effect is usually partial and less consistent.

For example, vaccines such as Cervarix and Gardasil generate immune responses against some high-risk HPV strains such as HPV-16 and -18. Because of structural similarities in the L1 capsid proteins, the antibodies generated can sometimes recognize and bind to genetically related types such as HPV 31, 33, or 45. This phenomenon was observed more strongly with Cervarix, probably due to its particular adjuvant system, which can stimulate a broader immune response [44,45].

Cross-immunity provides limited protection because it is weaker, less durable, and less predictable than protection against the HPV types directly included in the vaccine. This is one of the reasons why the new Gardasil 9 was developed—it explicitly includes additional HPV types (31, 33, 45, 52, and 58) that were previously only partially covered by cross-reactivity [46].

A study conducted in Fiji [34] in 200 women explored this phenomenon by dividing participants into four groups that received zero, one, two, or three doses of the tetravalent vaccine [34]. Seroconversion was monitored, highlighting a dose-dependent increase in joint protection and cross-reactivity. The results are summarized in Table 4.

As shown in the table, antibody responses against both vaccine-targeted types (HPV-6, 11, 16, 18) and non-targeted types (HPV-31, 33, 45, 52, 58) improved with each additional dose. Even though this data is only qualitative, it highlights not only the necessity of completing the full vaccination schedule but also the potential for partial cross-protection against non-vaccine HPV strains [47].

There has also been studies about cross-priming, a way of the immune system to generate immune memory and induce cross-reactive antibody responses. Cross-priming relies on dendritic cells. Thei take the VLPs (which are non-infectious antigenic particles), and present them to naïve CD8+ T-cells [20]. This leads to the creation of CD8+ memory cells that, on a possible future contact with the virus, a similar strain, or another dose of vaccine, determine a potent immune response. In a research published in 2019, patients who received a dose of either bivalent or quadrivalent vaccine and naïve patients were given a shot of nonavalent vaccine. After this, they were screened for titers of antibodies against HPV-31/33/45/52 [20]. The GMT was significantly higher in vaccinated patients than in the group of naïve patients. This indicates that a “pre-programmed” immune system is capable of mounting better immune responses [20].

#### 3.2.3. Immune Response After Vaccination

The consensus is unequivocal: vaccination provides the most effective protection against both new and persistent HPV infections. By inducing durable antibody levels, vaccination has protective capacity against subsequent malignancies. More so, it decreases the morbidity and mortality associated with HPV infection, highlighting its role in public health protection [48].

**Figure 2:**
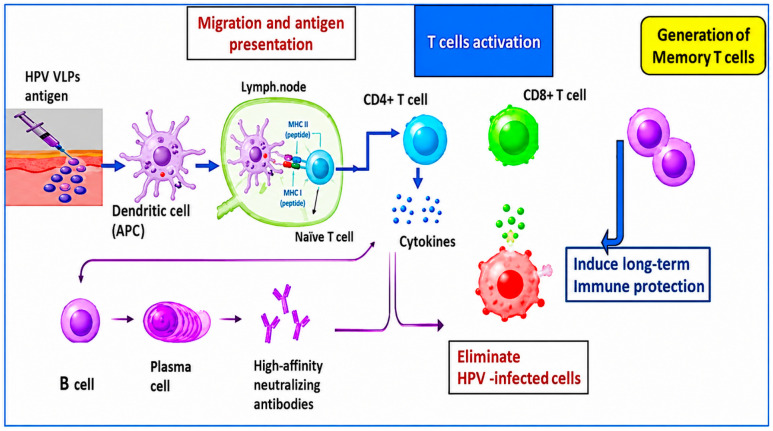
The immune response induced by HPV virus-like particle (VLP) antigens. Following administration, HPV VLPs are captured by dendritic cells (antigen-presenting cells, APCs), which migrate to the lymph node and present antigenic peptides via MHC I and MHC II molecules to naïve T cells. This leads to activation of CD4+ helper T cells and CD8+ cytotoxic T cells. CD4+ T cells secrete cytokines that support immune signalling and activation, while CD8+ T cells mediate the elimination of HPV-infected cells. In parallel, B cells are activated and differentiate into plasma cells that produce high-affinity neutralising antibodies.

HPV vaccination elicits a strong humoral response, associated with a sustained cellular immune response, contributing to long-lasting protection. HPV vaccination induces a coordinated and robust cell-mediated immune response, essential for the development of long-term protection. After intramuscular administration, vaccine antigens (virus-like particles, VLPs) are taken up by antigen-presenting cells (APCs), particularly dendritic cells, at the injection site. These cells process the antigen and migrate to regional lymph nodes, where they present HPV-derived peptides via MHC molecules to naive T lymphocytes [49]. This interaction leads to the activation and differentiation of CD4+ helper T cells, which play a central role in orchestrating the immune response by secreting cytokines that support B-cell activation, proliferation and antibody production. In parallel, CD8+ cytotoxic T cells can be activated, contributing to the recognition and elimination of HPV-infected cells [21]. Importantly, the vaccine also induces the formation of memory T cells, which persist in the long term and enable a rapid, efficient immune response upon subsequent exposure to the virus. (Figure 2) [46].

A Dutch study conducted in 2022 investigated the cellular immune response following HPV vaccination in 20 women who were seronegative at baseline [50]. Each participant received three doses of bivalent or nonavalent vaccine, and blood samples were collected at different time points. No significant long-term difference was found in subjects who received the bivalent or nonavalent formulations [50].

In the immediate post-vaccination period, monocytes dominated the peripheral blood response, peaking within the first three days before declining. Neutrophils subsequently became the principal leukocyte population in circulation [50]. A slight decline in T-cell count was noted in the first three days, after which they tend to rebound, highlighting their role in inducing the humoral immune response. B cells tended to increase a week after each dose of the regimen, coinciding with the onset of antibody production. By half a year, IgG antibodies become the most important immune mechanism, offering durable protection [50].

#### 3.2.4. Degree of Protection and Antibody Kinetics

Vaccination against HPV infection induces high levels of neutralizing antibodies, significantly exceeding those generated by natural infection, and provides strong protection against vaccine-type HPV infections. Antibody titers peak shortly after completion of the vaccination schedule, followed by a gradual decline and stabilization into a long-term plateau, consistent with durable immune memory. Despite decreasing titers, protection remains sustained over time, supported by the presence of memory B cells and rapid anamnestic responses upon re-exposure [51].

One of the earliest large-scale research studies emphasizing vaccine efficacy was conducted in Costa Rica [16,52], involving 7466 women who received one, two, or three doses of the bivalent HPV vaccine. Antibody titers were tracked for up to 10 years. Although titers were highest in women who received three doses, no statistically significant difference in protection was observed. Importantly, participants who received only a single dose did not show increased rates of cervical lesions or malignancies compared to those who completed the full regimen [16,52].

These findings were later upheld by studies on the quadrivalent vaccine [22], with similar dosing regimens and 10-year follow-up. Antibody kinetics revealed a peak a few months after immunization, followed by a gradual decline and stabilization around 18 months after the final dose [22]. Even though antibody levels after a single dose were lower than those after two or three doses, they remained protective. This is explained by the relatively slow epithelial penetration of HPV, which allows antibodies—even at lower concentrations—to act effectively [22].

Similarly, a study conducted in Tanzania [17] compared the impact of receiving either one, two, or three doses of the nonavalent or bivalent vaccine. Of the 950 individuals initially enrolled, 922 completed the trial. They randomly received one, two, or three doses of vaccine, and after that, seropositivity was tested at the 2-year mark [17]. For HPV-16, 99% of those who received a single dose were seropositive, compared with 100% in the two- and three-dose groups. Similar findings were reported for HPV-18, with seropositivity rates of 98% and 100%, respectively [17].

On the basis of such results [17,23], the World Health Organization recommended that vaccination programs include at least one dose, as even a single administration provides protection far superior to that achieved through natural immunity. They are also supporting the feasibility of dose-sparing strategies in resource-constrained settings [17,23].

##### Protective Role of IgA Antibodies

After vaccination against HPV infection, IgA antibodies contribute to immune protection primarily at mucosal surfaces, which are the main entry sites for the virus [53]. Although HPV vaccines such as Gardasil 9 are administered intramuscularly and primarily elicit a strong systemic IgG response, they also stimulate the production of IgA antibodies detectable in cervical and other mucosal secretions. These IgA antibodies help neutralize the virus by preventing its attachment and entry into epithelial cells, thereby acting as an early line of defense. While their role is secondary to IgG in long-term protection, IgA antibodies enhance local immunity and contribute to the vaccine’s overall effectiveness in preventing HPV infection [24]. In the study conducted by R. Murillo it was shown that IgA antibodies, abundant in the vaginal and cervical epithelium, offer strong local protection [42]. Their concentration begins to decline approximately 7 months post-vaccination and is influenced by elements like the viscosity of vaginal and cervical secretions. Notably, these antibodies decrease HPV infections during this period, decreasing the demand for plasma-derived antibody extravasation [42].

##### Post-Infection Vaccination Utility

HPV infection is known to persist latently in cervical epithelial cells, with the potential for recurrence. Before the introduction of vaccination, achieving an HPV-free status was difficult for previously exposed individuals. A Dutch study [18] followed antibody titers before and after bivalent vaccination in women aged 14–27 years, with monitoring extending up to 10 years. The vaccine demonstrated high efficacy against persistent infections with HPV-16 and HPV-18 (95.8%, 95% CI), strong protection against new infections (84.5%), and moderate efficacy against cross-reactive strains (64.6%) [18]. Antibody titers remained stable after 24 months and persisted for up to a decade, with most women achieving HPV-free status within 18 months of the final dose [18].

Similarly, a study in Poland [25] evaluated 60 women for HPV DNA, of whom 12 tested positive, including six with strains covered by the nonavalent vaccine. Of the cohort, 51 subsequently received the nonavalent vaccine [25]. Six months after vaccination, assessments of antibody titers, HPV DNA detection, Pap smears, and conization outcomes confirmed the vaccine’s ability not only to prevent new infections but also to contribute to the clearance of existing lesions [25].

Even after HPV exposure, vaccination acts as a preventive “upgrade” of immunity, improving protection against reinfections, new infection with additional types, recurrence of disease.

## 4. Vaccine Immunogenicity

The immunogenicity of HPV vaccines is a key advantage in the prevention of HPV infection and its associated malignancies. Immunogenicity varies across vaccine formulations. A small study of 52 women [54] tracked neutralizing antibody titers over a period of 12 months. Results indicated higher immunogenicity for the bivalent vaccine compared to quadrivalent and nonavalent formulations [54]. The comparative results, as demonstrated at 1, 6, and 12 months, are shown in Table 5.

Only 10 women received the bivalent vaccine, with 7 attending the 1-month follow-up and 3 at 6 months. Despite the small sample size, the bivalent vaccine showed increased immunogenicity, most likely attributed to higher antigenicity and narrower strain coverage. In contrast, the nonavalent formulation showed delayed seroconversion (for HPV 18), indicating the broader strain distribution and lower antigenic load [54].

Similar findings were reported in the United Kingdom [55], where a study compared 727 individuals split into three groups: women receiving three doses of the bivalent vaccine, women receiving two or three doses of the quadrivalent vaccine, and men receiving two doses of the quadrivalent vaccine. Seropositivity was higher with the bivalent vaccine, reaching 94% for HPV-16 and 86% for HPV-18 [55]. In addition to efficacy outcomes, the researchers proposed standardized cut-off values for antibody titers, aiming to harmonize comparisons across studies and vaccine types. These thresholds are 171 UI/mL for anti-HPV-6 antibodies, 317 UI/mL for anti-HPV-11 antibodies, 47 UI/mL for anti-HPV-16 antibodies and 26 UI/mL for anti-HPV-18 antibodies, respectively. It is unclear whether these values have become accepted and used in practice [55].

Overall, HPV vaccines induce a rapid, strong, and long-lasting immune response, producing high levels of neutralising antibodies and immune memory more superior to natural infection in preventing future disease.

## 5. Immune Response in Mixed Cohorts

The analysis of immune response in mixed cohorts refers to the evaluation of immunological outcomes within heterogeneous populations that include individuals with varying demographic and clinical characteristics, such as age, sex, prior exposure to HPV infection, and vaccination status. This approach is particularly relevant in HPV research, where populations are rarely uniform and may comprise both previously infected and HPV-naïve individuals, as well as vaccinated and unvaccinated subjects. By assessing parameters such as antibody titers, seroconversion rates, and the durability of immune responses across these subgroups, mixed cohort analyses provide a more comprehensive understanding of immune variability and vaccine performance in real-world settings. Furthermore, such studies enable direct comparisons between natural and vaccine-induced immunity, helping to identify factors that influence immunogenicity, including age, immune status, and prior viral exposure. Ultimately, evaluating immune responses across mixed cohorts supports the development of more effective vaccination strategies and evidence-based public health recommendations to reduce the burden of HPV-related diseases.

A study conducted in Alaska assessed vaccine-induced antibodies in a mixed cohort of 227 participants [19]. Each individual received 2 doses of nonavalent vaccine, the second given at 5 to 12 months after the first. Follow-up attendance varied: 205 participants were screened one month after the first dose, 197 one month after the second dose, 172 at one year, and 145 at three years [19]. Only 107 completed all the screenings. The results about seropositivity rates and the Geometric Mean Concentration (GMC) of antibodies are shown in Table 6 [19].

As shown above, seropositivity rates increase after the second dose is received, reaching 100% across all vaccinal strains one month after completion of the regimen. At the three-year mark, antibody presence stabilised, with a slight decline for HPV-45 [19]. GMC values declined over time (Cl = 95%), still remaining at protective values, according to findings in the UK cohort shown above. Minor sex-related differences were noted: men generally exhibited lower antibody titers, though seropositivity rates were comparable to those of women [19].

Some similar data comes from a Dutch study [26] that followed 121 participants (56 women and 55 men) who received two doses of the bivalent HPV vaccine. All participants present at follow-up demonstrated seroconversion for both HPV-16 and HPV-18. Antibody concentrations were expressed in local units (LU/mL) and monitored at one month and three years, with a subset of participants reassessed at 7.5 years [26]. The results are summarized in Table 7.

Despite the relatively small cohort and the missing standardized units across studies, the results correspond with previous reports of time-dependent antibody decline. Notably, men exhibited higher antibody levels at both follow-ups, but the same kind of evolution pattern. These differences may reflect the specific use of the bivalent vaccine, sex-related immunological variation, or individual heterogeneity in immune response [26]. For the few participants reassessed at 7.5 years (number not specified), antibody concentrations remained similar to those measured at three years, suggesting plateau-like kinetics in long-term antibody persistence.

A study conducted in the United States evaluated vaccine success in a 1108 mixed-person cohort (993 men and 115 women), with a history of more than one sexual partner [56]. Of the 1108, only 40 ever received an HPV vaccine. Oropharyngeal secretions, genital samples, and blood were analyzed for viral particles and HPV antibodies. The results showed that 248 individuals carried an oncogenic HPV strain, most commonly HPV-16, which accounted for less than half of the detected cases [56]. Antibodies against oncogenic HPV types were found in 179 participants, with anti-L1, anti-E6, and anti-E7 being the most common. Notably, the 40 vaccinated individuals exhibited significantly higher antibody counts compared with the unvaccinated group, pointing to the protective effect of vaccination even in populations with elevated sexual exposure [56].

## 6. Immune Memory

The immune memory plays a critical role in the long-term effectiveness of vaccination against HPV infection. A cohort of 150 individuals received three doses of the nonavalent HPV vaccine, with follow-up evaluations performed at day 2, month 2, month 6, and month 60 [27]. Antibody titers peaked at six months following the third dose, then gradually declined between months 7 and 36, eventually stabilizing into a plateau phase. Notably, at the 60-month mark, before booster administration, between 77.5% and 100% of vaccine-targeted HPV strains remained covered by detectable antibodies [27]. Participants then received a booster dose, followed by evaluations at day 7 and day 28 post-booster. At day 7, 99% of individuals were seropositive for all vaccine strains, and by day 28, seropositivity reached 100% across all strains. This increase in antibody levels following re-exposure provides strong evidence for the persistence of functional immune memory, likely mediated by long-lived memory B cells, and supports the sustained immunogenicity and long-term protective efficacy of HPV vaccination [27].

## 7. Vaccination and Local Lesions

Vaccination in the context of existing local lesions associated with HPV infection represents an important adjunct strategy in clinical management, although it does not exert a direct therapeutic effect on established disease. Current evidence indicates that HPV vaccines do not eliminate pre-existing infections or induce regression of established lesions, such as cervical intraepithelial neoplasia or genital warts. However, vaccination in individuals with prior or ongoing HPV-related lesions may provide significant benefits by enhancing systemic immune protection against other oncogenic HPV to which they might be naïve.

Findings about the local impact of HPV vaccination were provided by several studies. One multi-continental investigation followed a mixed cohort of 1272 people (301 men and 971 women) who received three doses of nonavalent vaccine [28]. Blood samples and genital secretions, including Pap smears, were collected every six months for up to 126 months. At the final evaluation, seropositivity was 95% across all antibody types, although only 81% for neutralising antibodies [28]. Importantly, there were no recorded cases of high-grade squamous intraepithelial lesions (HSIL), condyloma, or genital cancer. The rate of persistent infections was low, at 54.6/10,000/year for men and 52.4/10,000/year for women [28].

Similar results were reported by scientists from Canada, the US, and Colombia [29]. In a cohort of 3253 people (men and women), the quadrivalent vaccine was administered, with genital samples being collected every 6 months over a period of 10 years. At the time of the final evaluation, only a case of condyloma was recorded, and also a case of intraepithelial neoplasia grade 2–3 [29]. All other lesions, if present, were classified as low-grade squamous intraepithelial lesions (LSIL). The risk-reduction was approximated for each type of lesion > 99.99% per protocol population (Cl = 95%) [29].

Another study conducted by American scientists in Kenya compared HPV vaccines with an unrelated control vaccine [30]. A total of 2275 women were separated into 3 groups: 758 received one dose of nonavalent HPV vaccine, 760 one dose of bivalent formulation, and 757 the antimeningococcal vaccine. After 6 months, vaginal secretions and PAP smears were collected. Only two new HPV infections were detected among women who received either HPV vaccine, compared with 36 new infections in the control group [30]. Efficacy was approximated at 97.5% for both the bivalent and nonavalent vaccines (*p* < 0.0001), underlining their strong protective effect [30].

These findings [28,29,30] underscore that vaccination not only maintains long-term antibody responses but also translates into meaningful reductions in lesion incidence and persistent infection, reinforcing its role in cancer prevention.

## 8. Vaccination in HPV-Positive Women

A prospective study evaluated the combined effect of HPV vaccination and LEEP-conization in a cohort of sixty HPV-positive patients [57]. The study group included fifty-one women vaccinated with the nine-valent formulation, while nine remained unvaccinated as controls. No differences were observed between groups in age and or obstetric history [57]. It should be noted that the impact of LEEP-conization alone on the disappearance of HPV infection was not mentioned. Although, when combined with the 9v-HPV vaccine, it leads to high curative efficacy [57].

Persistent HPV infection was significantly less frequent in the vaccinated group (23.5%) compared with controls (88.9%, *n* = 8; *p* < 0.001). When only nonavalent-covered types (6, 11, 16, 18, 31, 33, 45, 52, 58) were considered, persistence was even lower (11.8%, *n* = 6) versus 66.7% (*n* = 6) in the control group (*p* = 0.001) [57]. Antibody levels were markedly higher in vaccinated patients, with reactive antibody responses detected in all vaccinated individuals compared to only one-third of controls (33.3%, *n* = 3; *p* < 0.001). This evidence suggests that vaccination in HPV-positive women enhances antibody responses and markedly reduces the persistence of infection, supporting its possible role as a secondary preventive measure [57].

## 9. HPV Vaccine and Respiratory Papillomatosis

Beyond its genital implications, HPV infection carries important risks for the development of upper airway lesions, including oropharyngeal papillomatosis. Recurrent respiratory papillomatosis (RRP) is a rare but potentially severe disease caused primarily by low-risk types of Human papillomavirus infection, especially HPV types 6 and 11. It is characterized by the growth of benign papillomas in the respiratory tract, most commonly affecting the larynx, and can lead to voice changes, airway obstruction, and the need for repeated surgical interventions. Prophylactic vaccines such as Gardasil 9 protect against these types and have been associated with a significant reduction in juvenile RRP incidence, primarily by preventing maternal HPV infection and vertical transmission during childbirth [58].

In Czechia, a study of 50 individuals with respiratory papillomatosis (13 newly diagnosed and 37 with pre-existing disease) evaluated the response to a full regimen of the quadrivalent vaccine [59]. At baseline, 30 participants underwent biopsy, and 48 provided pharyngeal smears. HPV-6 was detected in 24 biopsies, HPV-11 in four, and HPV-16 and HPV-18 in one case each. Follow-up evaluations were conducted at 1 month, 1 year, and 5 years [59]. After one month, all participants exhibited HPV antibody levels approximately 100-fold higher than baseline. At the final five-year evaluation, antibody levels remained tenfold higher than baseline. Clinically, vaccination achieved an efficacy of 28.6% in preventing recurrences and 9.5% in preventing disease progression. Although progression continued in most cases, it occurred at a slower rate [59]. Despite the small cohort size, the data illustrate the potential of prophylactic HPV vaccination as an important means of combating upper airway lesions and reducing or slowing progression to malignancy [59].

## 10. Interactions and Risks

Vaccination against HPV infection is generally well tolerated and associated with a strong safety profile, with minimal clinically significant drug or vaccine interactions. Prophylactic vaccines such as Gardasil 9 can be co-administered with other routine immunizations without compromising immunogenicity or safety. The most commonly reported adverse effects are mild and transient, including injection-site reactions, headache, and low-grade fever. Rare events such as syncope and hypersensitivity reactions, including anaphylaxis, have been reported but occur at very low frequencies. Immunocompromised individuals may exhibit reduced immunogenic responses, although vaccination remains safe and recommended. Importantly, HPV vaccines are non-live and therefore cannot cause infection or infertility, and no consistent evidence supports an association with autoimmune diseases. Vaccination during pregnancy is not routinely recommended, although inadvertent exposure has not been linked to adverse outcomes.

A study conducted in Canada in 2023 evaluated the immune response of vaccinated patients receiving immunosuppressant treatment [31]. The cohort included 33 individuals aged 12–19, 16 healthy controls, and 17 transplant recipients (7 kidney and 10 liver). Six of the kidney transplant recipients were on triple immunosuppressive therapy, while nine of the liver transplant recipients were on at least one immunosuppressant [31]. All participants received a full regimen of the quadrivalent vaccine, with blood samples collected at multiple time points. The results they found are shown in Table 8.

As the table shows, in kidney transplant patients, who typically receive more intense immunosuppressive therapy, seropositivity tends to be significantly reduced compared to liver transplant recipients or healthy individuals (it is important to note that the kidney transplant recipient who received less immunosuppressive therapy had higher antibody levels) [31]. Cross-reactivity appeared unaffected, with only vaccine-type-specific antibodies (HPV-6, 11, 16, 18) exhibiting decreased levels. Although the cohort size was limited, this evidence indicates that HPV vaccination is useful in immunosuppressed populations. Even when antibody titers are lower, vaccination provides measurable immune responses and should be considered as part of preventive care in transplant recipients [31].

A study in China assessed some of the risks of HPV vaccination in people with immune disregulations [32]. Although HPV vaccines are characterized by a very high safety profile, rare but severe adverse reactions have been reported in specific populations. In most cases, vaccination was well tolerated, but reactions like autoimmune encephalitis were observed in subsets of patients. Typically, autoimmune diseases prompt the formation of anti-glutamic acid decarboxylase 65 antibodies [32]. These antibodies target neural tissue and may lead to sensory or motor deficits, which may persist indefinitely. Overall, HPV vaccination continues to be safe and effective, but clinicians must stay alert when administering it to immunologically at-risk groups [32].

## 11. Discussion

Through this review, we wanted to determine whether naturally acquired immunity provides protection comparable to vaccine-induced immunity against HPV infection and its complications. The available evidence consistently indicates that it does not. Natural immunity is highly variable, type-specific, and often insufficient to prevent infection [7,42], whereas vaccination induces substantially higher and more durable neutralizing antibody titres, which correlate with longer-lasting protection. Given these findings, a clear action would be to integrate single-dose HPV vaccination into national cancer prevention plans. This strategic inclusion could significantly enhance the public health response by offering scalable and impactful protection against the virus, thereby helping to curb HPV-related complications more effectively.

The cellular and humoral information highlighted in this paper demonstrates why vaccine-induced immunity regularly exceeds naturally acquired immunity. The cellular events occurring after vaccination show a coordinated immune activation pattern. In the Dutch study [50], monocytes dominated the initial response in the first three days, followed by a shift toward neutrophil predominance. This rapid innate immunity activation is typical of vaccines and sets the stage for responsive immunity. The transient decline and subsequent rebound of T-cell populations further highlight their central role in facilitating B-cell activation [50]. The rise in B-cell numbers approximately one week after each dose corresponds with the onset of antibody production, eventually resulting in the establishment of neutralising IgG antibodies by six months. This progression, from innate activation to durable IgG-mediated protection, contrasts with the variable and often weak immune responses generated after natural HPV infection [50].

Antibody kinetics reinforce this superiority. Large-scale studies from Costa Rica [16,52] and subsequent quadrivalent vaccine cohorts [22] show that antibody titers peak shortly after immunization and later stabilize, with protection remaining efficient for at least 10 years. Importantly, the degree of protection does not correlate linearly with antibody quantity. Even though single-dose recipients display diminished titers compared to those receiving two or three doses, clinical outcomes remain equivalent. The explanation is HPV’s low capacity to penetrate the epithelium, which allows even low concentrations of neutralizing antibodies to intercept the virus before it reaches the basal layers. This advantage explains why single-dose schedules maintain high efficacy despite lower antibody levels.

Evidence from Tanzania [17] further supports the rationale for dose-sparing strategies. Seropositivity rates for HPV-16 and HPV-18 remained exceptionally high (99–100%) regardless of whether participants received 1, 2, or 3 doses of the nonavalent or bivalent vaccine. This evidence, consistent throughout continents and vaccine formulations, demonstrates that a single dose reliably induces a protective immune response [17]. On this basis, the World Health Organization now endorses at least one dose as sufficient for meaningful protection, a recommendation with major effects on worldwide immunization programs, particularly in resource-constrained settings.

Across vaccine formulations and populations, the evidence reliably shows that HPV vaccination induces strong, durable, and clinically important immune responses. Immunogenicity varies by formulation, with the bivalent vaccine generating the highest neutralizing antibody titers in several cohorts. This likely indicates its narrower antigenic focus and higher antigen load. In contrast, the nonavalent vaccine shows slightly delayed seroconversion for certain types, such as HPV-18, due to wider strain allocation, yet still achieves strong protection.

Mixed-cohort studies reinforce this evidence. In Alaska [19], two-dose nonavalent vaccination achieved 100% seropositivity across all strains after regimen completion, with antibody levels stabilizing over 3 years and a gradual decline in GMC. Similar long-term patterns were observed in Dutch [26] participants receiving the bivalent vaccine, where antibody concentrations remained detectable even 7.5 years post-vaccination. Minor sex-related differences in antibody magnitude were noted, but seroconversion rates remained high. Booster administration triggers a rapid response, demonstrating preserved immune memory and supporting the durability of vaccine-induced protection.

These immunological advantages translate directly into clinical outcomes. Large, multi-continental cohorts vaccinated with the nonavalent or quadrivalent vaccines show low rates of persistent infection and virtually no high-grade lesions or genital cancers over 10–12 years of follow-up [28,29,30]. Even in HPV-positive women, vaccination markedly reduces persistence and strengthens the antibody response, supporting its role as a secondary preventive measure. Beyond the genital tract, vaccination also shows potential to reduce recurrence and slow progression of respiratory papillomatosis, underscoring its broader protective potential [46].

Evidence from special populations shows that while HPV vaccines maintain an excellent safety profile, immune status can affect both immunogenicity and adverse reactions. In the Canadian cohort of transplant recipients [31], liver-transplant patients mounted antibody responses comparable to healthy controls, whereas kidney-transplant recipients—who were typically on more intensive immunosuppressive therapy—showed reduced seropositivity and lower antibody titres [31]. Despite this diminishment, cross-reactivity remained intact, and all groups demonstrated measurable immune responses, supporting vaccination as a valuable preventive measure even in heavily immunosuppressed individuals [31].

Conversely, data from China [32] highlight that rare but severe adverse events may occur in patients with underlying autoimmune diseases. Autoimmune encephalitis developed post-vaccination, associated with anti-GAD65 antibodies, was observed in a small subset of individuals [32]. Although such reactions are exceptional, they point out the importance of careful clinical assessment and monitoring in patients with known autoimmune tendencies. Overall, these results support that HPV vaccines are safe and broadly effective throughout different populations, but immunological vulnerability warrants individualized consideration [32].

HPV vaccination induces a markedly stronger and more durable immune response compared with natural infection [5,60]. Vaccine-induced immunity elicits peak neutralizing antibody titers exceeding 3000–10,000 mMU/mL against L1 capsid proteins—orders of magnitude higher than the 100–500 mMU/mL typically seen post-natural exposure—while providing broader cross-protection against non-vaccine high-risk genotypes like HPV 31, 33, 45, 52, and 58 [5,61].

In contrast, natural immunity remains weak, type-specific, and short-lived, with 70–90% of infections clearing within 1–2 years but reinfection rates reaching 20–50% due to suboptimal memory B-cell responses [15,62]. Longitudinal data confirm vaccine efficacy: Gardasil and Cervarix achieve 90–100% protection against HPV16/18-related cervical intraepithelial neoplasia grade 2+ (CIN2+), with real-world reductions in precancer incidence of 40–90% in vaccinated cohorts [5,61].

Taken together, the evidence demonstrates that vaccine-induced immunity is consistently stronger, more durable, and more clinically protective than natural immunity. Across healthy, immunosuppressed, and high-risk populations, HPV vaccination provides important reductions in infection, persistence, and lesion development, firmly establishing it as a key element of global cancer prevention.

To summarize the differences detailed in this review, a direct comparison between naturally acquired and vaccine-induced immunity, highlighting key parameters such as antibody response, duration, and clinical efficacy, is presented in Table 9.

## 12. Conclusions and Future Perspectives

While current HPV vaccines are inherently prophylactic rather than therapeutic, they induce a robust and durable immune response that significantly outperforms natural immunity, leading to a profound reduction in precancer incidence. However, realizing the full public health potential of these vaccines requires addressing persistent barriers to global access, particularly in low- and middle-income countries, through cost-reduction strategies and simplified dosing schedules. Moving forward, continued research is essential to develop next-generation vaccines with broader genotype coverage and potential therapeutic effects, as well as to define clear correlates of protection across diverse populations. Ultimately, integrating widespread vaccination with comprehensive screening programs remains the most effective strategy to reduce and potentially eliminate the global burden of HPV-related cancers.

## Figures and Tables

**Table 1:** Most prominent studies explicitly detailed in this review, highlighting their methodologies, cohort sizes, and geographical locations.

Author [Reference]	Year	Geographical Location	Methods/Cohort Size	Main Focus of the Study
Rosillon et al. [16]	2019	Netherlands	Cohort study, thousands of women near menopause	Natural immunity and protection against persistent infection
Yao et al. [17]	2022	China	Serological analysis, 978 unvaccinated women	Natural neutralizing vs. total IgG antibodies
Morais et al. [18]	2023	Canada	Seroprevalence assessment, 222 unvaccinated women	Baseline natural immunity and antibody types
Syrjänen et al. [19]	2022	Finland	Longitudinal serum analysis, 276 mother–infant pairs	Placental antibody transfer and early postnatal exposure
Porras et al. [20]	2020	Costa Rica	Large-scale trial, 7466 women, 10-year follow-up	Bivalent vaccine efficacy (1, 2, or 3 doses)
Watson-Jones et al. [21]	2022	Tanzania	Randomized trial, 950 participants, 2-year follow-up	Dose-sparing (1, 2, 3 doses) nonavalent vs. bivalent
Hoes et al. [22]	2023	Netherlands	Longitudinal monitoring (10 years), women aged 14–27	Bivalent vaccine efficacy against persistent infections
Pruski et al. [23]	2022	Poland	Clinical assessment, 60 women (12 HPV+), 6-month follow-up	Nonavalent vaccine administration post-infection
Panwar et al. [24]	2025	United Kingdom	Comparative serosurveillance, 727 individuals	Immunogenicity of bivalent vs. quadrivalent vaccine
Steinberg et al. [25]	2025	USA (Alaska)	Prospective mixed cohort, 227 participants, 3-year follow-up	2-dose nonavalent vaccine immunogenicity
D’Souza et al. [26]	2023	USA	Cross-sectional study, 1108 mixed-person cohort	Vaccine protection in high-sexual-exposure populations
Restrepo et al. [27]	2023	Multi-continental	Long-term follow-up (126 months), 1272 participants	Nonavalent vaccine impact on local lesions/persistence
Maldonado et al. [28]	2022	Canada, USA, Colombia	10-year follow-up, 3253 participants (men and women)	Quadrivalent vaccine efficacy on genital lesions
Barnabas et al. [29]	2022	Kenya	Randomized trial, 2275 women, 6-month follow-up	Single-dose nonavalent/bivalent vs. control vaccine
Pruski et al. [30]	2023	Poland	Prospective study, 60 HPV+ patients	9-valent vaccine combined with LEEP-conization
Smahelova et al. [31]	2022	Czechia	Clinical tracking (up to 5 years), 50 individuals with RRP	Quadrivalent vaccine for respiratory papillomatosis
Kitano et al. [32]	2023	Canada	Immunological assessment, 33 transplant/immunosuppressed	Quadrivalent vaccine immunogenicity in transplant recipients

**Table 2:** Seropositivity rates among commonly found strains.

HPV Type	Seropositivity Rate (%)	Notes
HPV 6	15.7	Highest seropositivity rate among tested types
HPV 16	10.2	Higher than HPV 18
HPV 18	—	Lower than HPV 16 (exact % not specified)
HPV 33	1.6	Low seropositivity
HPV 58	3.1	Low seropositivity

**Table 3:** Antibody types and roles in malignancy prevention.

Antibody Type	Relative Abundance	Role in Malignancy Prevention
IgG anti-L1	Most prevalent	Structural protein not directly linked to malignancy prevention
IgG anti-E7	Moderate	Considered effective in preventing lesion malignancy
IgG anti-E6	Rare	Also effective in preventing lesion malignancy

**Table 4:** Protection and cross-reactivity by dose.

PROTECTION AND CROSS-REACTIVITY BY DOSE	0 DOSES	1 DOSE	2 DOSES	3 DOSES
HPV-6	/	LOW	MODERATE	HIGH
HPV-11	/	LOW	MODERATE	HIGH
HPV-16	LOWEST	LOW	MODERATE	HIGH
HPV-18	LOWEST	LOW	MODERATE	HIGH
HPV-31	/	LOWEST	LOW	MODERATE
HPV-33	/	LOWEST	LOW	MODERATE
HPV-45	/	LOWEST	LOW	MODERATE
HPV-52	/	LOWEST	LOW	MODERATE
HPV-58	/	LOWEST	LOW	MODERATE

**Table 5:** Comparison of antibody titres induced by different vaccine types and follow-up periods. GMT: Geometric Mean Titre.

FOLLOW UP	NEUTRALISING ANTIBODY	NONAVALENT VACCINE		QUADRIVALENT VACCINE		BIVALENT VACCINE		*p*
		MEAN	GMT	MEAN	GMT	MEAN	GMT	
**1 MONTH**	HPV-16	13,486	9545	8225	8222	8705	8702	0.5082
	HPV-18	7132	4583	4450	4449	7715	7685	0.0896
**6 MONTHS**	HPV-16	4626	3580	7680	7612	10,129	9849	<0.0001
	HPV-18	8737	7740	4360	4267	8357	8138	0.0004
**12 MONTHS**	HPV-16	6359	4638	6812	4758			0.2482
	HPV-18	11,388	7181	4010	2271			0.0006

**Table 6:** Variation in seropositivity and antibody concentration in time.

HPV STRAIN	6 Months After First Dose (N = 205)		1 Month After Second Dose (N = 197)		1 Year After Second Dose (N = 172)		3 Years After Second Dose (N = 145)	
	Seropositivity	GMC (UI/mL)	Seropositivity	GMC (UI/mL)	Seropositivity	GMC (UI/mL)	Seropositivity	GMC (UI/mL)
6	99%	59	100%	4535	100%	469	100%	220
11	99%	42	100%	3727	100%	374	100%	171
16	>99%	22	100%	1781	100%	219	100%	94
18	94%	13	100%	635	96%	62	96%	27
31	99%	26	100%	1554	98%	176	99%	88
33	98%	36	100%	2540	99%	274	100%	123
45	85%	8	100%	462	95%	45	92%	25
52	99%	96	100%	2960	99%	368	100%	184
58	99%	93	100%	3483	100%	435	100%	207

GMC: Geometric Mean Concentration.

**Table 7:** Anti HPV antibody variation through time.

HPV TYPE	WOMEN		MEN	
	1 Month After Dose 2	3 Years After Dose 2	1 Month After Dose 2	3 Years After Dose 2
**HPV-16**	6000 LU/mL	482 LU/mL	9069 LU/mL	Significantly higher
**HPV-18**	6606 LU/mL	159 LU/mL	4215 LU/mL	Significantly higher

**Table 8:** Seropositivity and antibody concentrations (MU = Merck Units) in study group.

STUDY GROUP	SEROPOSITIVITY RATE	ANTIBODY CONCENTRATION (MU/mL)
Control (N = 16)	100%	638.8–4391.6
Liver transplant (N = 10)	100%	569.3–3097.3
Kidney transplant (N = 7)	50–75%	8.6–42.4

**Table 9:** Summary of the differences detailed in this review.

Feature	Naturally Acquired Immunity	Vaccine-Induced Immunity
**Primary Immune Response**	Dominated by cellular responses; slow, highly variable, and often incomplete seroconversion.	Coordinated robust humoral and cellular responses (activation of memory T and B cells); rapid and nearly 100% seroconversion.
**Antibody Levels**	Low and weak neutralizing antibody titers (typically 100–500 mMU/mL).	Significantly higher neutralizing IgG antibody titers (exceeding 3000–10,000 mMU/mL).
**Duration of Protection**	Short-lived; memory B-cell responses are suboptimal, leading to high reinfection rates (20–50%).	Highly durable; antibody levels stabilize into a long-term plateau, persisting for ≥15 years without the need for boosters.
**Scope of Protection**	Strictly type-specific (protects only against the infecting strain).	Broad protection against targeted high-risk and low-risk strains, with proven cross-protection against genetically related non-vaccine strains.
**Clinical Efficacy**	Unreliable; frequently insufficient to prevent initial infection, persistent infection, or progression to malignancy.	Consistently superior; provides 90–100% protection against high-grade lesions (e.g., CIN2+) and significantly reduces viral persistence even in already infected individuals.

## Data Availability

No new data were created or analyzed in this study. Data sharing is not applicable to this article.

## Abbreviations

The following abbreviations are used in this manuscript:

**APCs**	Antigen-presenting cells
**CI/Cl**	Confidence Interval (mentioned as 95% CI or Cl = 95%)
**CIN**	Cervical intraepithelial neoplasia (mentioned in the text as CIN III or CIN2+)
**DNA**	Deoxyribonucleic acid
**GMC**	Geometric Mean Concentration
**GMT**	Geometric Mean Titer (found in the antibody tables)
**HPV**	Human Papillomavirus
**HSIL**	High-grade squamous intraepithelial lesions
**IgA, IgG, IgM**	Immunoglobulin A, G, and M (types of antibodies)
**IU/mL**	International Units per milliliter
**LEEP**	Loop electrosurgical excision procedure (mentioned in the text as “LEEPconization”)
**LSIL**	Low-grade squamous intraepithelial lesions
**LU/mL**	Local units per milliliter
**MHC**	Major histocompatibility complex (e.g., MHC I and MHC II)
**MU/mL**	Merck Units per milliliter
**NK (cells)**	Natural killer cells
**RRP**	Recurrent respiratory papillomatosis
**VLPs**	Virus-like particles
